# The Navigation Guide Systematic Review Methodology: A Rigorous and Transparent Method for Translating Environmental Health Science into Better Health Outcomes

**DOI:** 10.1289/ehp.1307175

**Published:** 2014-06-25

**Authors:** Tracey J. Woodruff, Patrice Sutton

**Affiliations:** Program on Reproductive Health and the Environment, University of California, San Francisco, Oakland, California, USA

## Abstract

Background: Synthesizing what is known about the environmental drivers of health is instrumental to taking prevention-oriented action. Methods of research synthesis commonly used in environmental health lag behind systematic review methods developed in the clinical sciences over the past 20 years.

Objectives: We sought to develop a proof of concept of the “Navigation Guide,” a systematic and transparent method of research synthesis in environmental health.

Discussion: The Navigation Guide methodology builds on best practices in research synthesis in evidence-based medicine and environmental health. Key points of departure from current methods of expert-based narrative review prevalent in environmental health include a prespecified protocol, standardized and transparent documentation including expert judgment, a comprehensive search strategy, assessment of “risk of bias,” and separation of the science from values and preferences. Key points of departure from evidence-based medicine include assigning a “moderate” quality rating to human observational studies and combining diverse evidence streams.

Conclusions: The Navigation Guide methodology is a systematic and rigorous approach to research synthesis that has been developed to reduce bias and maximize transparency in the evaluation of environmental health information. Although novel aspects of the method will require further development and validation, our findings demonstrated that improved methods of research synthesis under development at the National Toxicology Program and under consideration by the U.S. Environmental Protection Agency are fully achievable. The institutionalization of robust methods of systematic and transparent review would provide a concrete mechanism for linking science to timely action to prevent harm.

Citation: Woodruff TJ, Sutton P. 2014. The Navigation Guide systematic review methodology: a rigorous and transparent method for translating environmental health science into better health outcomes. Environ Health Perspect 122:1007–1014; http://dx.doi.org/10.1289/ehp.1307175

## Introduction

There is an urgent unmet need to shorten the time between scientific discovery and improved health outcomes. Population exposure to toxic environmental chemicals is ubiquitous [Centers for Disease Control and Prevention (CDC) 2014; U.S. Environmental Protection Agency (EPA) 2013c], and adverse health outcomes associated with exposure to such chemicals are prevalent and on the rise (Newbold and Heindel 2010; Olden et al. 2011; U.S. EPA 2013c; Woodruff et al. 2010; World Health Organization and United Nations Environment Programme 2013). The health and economic benefits of translating scientific discoveries into actions to prevent harm and reap benefits have been clearly demonstrated. For example, global efforts to remove lead from gasoline have produced health and social benefits estimated at $2.4 trillion dollars annually (Tsai and Hatfield 2011); and the value of better air quality, including reductions in premature death and illness, and improved economic welfare and environmental conditions from the programs implemented pursuant to the Clean Air Act Amendments of 1990, will reach almost $2 trillion dollars in 2020 (U.S. EPA 2011). However, many potential benefits have been squandered due to delays in acting on the available science (European Environment Agency 2013). Because of deficiencies in the current regulatory structure for manufactured chemicals, a failure or delay in acting on the science means that exposure to toxic chemicals persists while evidence of harm mounts (Vogel and Roberts 2011).

Failing or delaying to take action to prevent exposure to harmful environmental chemicals is not an inconsequential or neutral policy choice. For example, the costs in 2008 to the U.S. health care system for treatment of childhood illnesses linked to toxic environmental exposures has been estimated to be > $76 billion (Trasande and Liu 2011). Failure to prevent even low-level environmental exposures can have large society-wide adverse consequences for health if exposures are ubiquitous (Bellinger 2012).

To the extent that science informs public policy to prevent harm, a robust method to synthesize what is known about the environmental drivers of health in a transparent and systematic manner is a necessary foundational step to making the science actionable. The body of science is voluminous, of variable quality, and largely unfamiliar to decision makers. Early warning signals of harm can be masked by the fragmented, complex, and at times, conflicting nature of the available information, undermining our capacity to act wisely. Yet, consistently applied and transparent rules and descriptors about how environmental health science is translated into strength of evidence conclusions have been lacking [Beronius et al. 2010; Gee 2008; National Research Council (NRC) 2009, 2011].

Today, methods of research synthesis prevalent in environmental health mirror that of clinical medicine > 40 years ago when the clinical sciences largely relied on a system of expert-based narrative reviews on which to recommend treatment decisions (Rennie and Chalmers 2009). In a landmark paper published in 1992 in the *Journal of the American Medical Association*, Antman et al. (1992) showed the superiority of systematic review methods by comparing expert opinion-based recommendations for treatment of myocardial infarction published in scientific reviews and clinical textbooks to statistical analyses of the combined results of randomized controlled trials. Antman et al. documented the lack of timely incorporation of experimental evidence into expert-based recommendations and showed that some expert reviews did not mention effective therapies, whereas others recommended therapies proven to be ineffective or even dangerous. From there, explicit approaches that harness expertise to a rigorous, transparent, and systematic methodology to evaluate a clearly formulated question were advanced, and are now embodied in prominent empirically demonstrated methods such as the Cochrane Collaboration (Higgins and Green 2011) and Grading of Recommendations Assessment, Development and Evaluation (GRADE) (Guyatt et al. 2008b). These methods are regularly relied on to inform decisions on billions of dollars of health care in order to achieve cost savings and better health outcomes (Fox 2010). Howells et al. (2012) estimated that utilization of systematic review and meta-analysis of the preclinical evidence (i.e., animal studies undertaken prior to human drug trials) could reduce the cost of developing drugs for treating stroke by $1.1–7.9 billion, the savings due to improving the validity of the evidence informing decisions on whether to advance drugs to clinical trials. It is anticipated that U.S. health care policy decisions will increasingly rely on systematic review methodologies; for example, health care reform legislation has allocated $1.1 billion dollars for comparative effectiveness research (CDC 2009).

The field of environmental health is now embarking on a similar journey. Reviews of the scientific evidence are as integral to decision making about exposure to environmental chemicals in national and local government agencies and industry as they are for making treatment decisions in clinical medicine. However, predominant approaches in use for evaluating the evidence in environmental health are > 30 years old, based on expert opinion, and with notable exceptions (Department of Health and Human Services 2006; National Toxicology Program 2013; U.S. EPA 2013b) generally do not provide strength of evidence summaries for outcomes other than cancer. Improved methods of risk assessment that better reflect our current understanding of the science have been articulated by the National Academy of Sciences in *Phthalates and Cumulative Risk Assessment: The Task Ahead* (NRC 2008) and in *Science and Decisions: Advancing Risk Assessment* (NRC 2009). Systematic approaches to evidence-based decision making that can improve our capacity to meet the needs of decision makers are also currently under way at the National Toxicology Program (Rooney et al. 2014) and under consideration at the U.S. EPA (NRC 2011, 2014a, 2014b). Described below are the results of the application of a novel method for systematic and transparent review in environmental health that demonstrate that such advances are not only desirable but within our grasp.

## Discussion

### Overview of the Navigation Guide Methodology

With the goal of expediting the development of evidence-based recommendations for preventing harmful environmental exposures, beginning in 2009 a collaboration of scientists and clinicians undertook the development of the Navigation Guide methodology for systematic review. The Navigation Guide methodology was developed by coupling the rigor of systematic review methods being used by the clinical sciences to the “bottom line” approach to research synthesis being used by the International Agency for Research on Cancer (IARC 2006). Features of systematic reviews used in clinical medicine encompass specifying an explicit study question, conducting a comprehensive search, rating the quality and strength of the evidence according to consistent criteria, and performing meta-analyses and other statistical analyses. IARC’s method allows for combining the results of human and nonhuman evidence into a single concise statement of health hazard (Woodruff et al. 2011).

As such, the Navigation Guide methodology translates the achievements of the past 20 years in evidence-based medicine into environmental health.

The Navigation Guide methodology involves four steps:

*Specify the study question:* Frame a specific question relevant to decision makers about whether human exposure to a chemical or class of chemicals or other environmental exposure is a health risk.
*Select the evidence:* Conduct and document a systematic search for published and unpublished evidence.
*Rate the quality and strength of the evidence:* Rate the quality of individual studies and the quality of the overall body of evidence based on prespecified and transparent criteria. The Navigation Guide methodology conducts this process separately for human and nonhuman systems of evidence. As a consequence, the methodology involves an additional step of integrating the quality ratings of each of these two streams of evidence. The end result is one of five possible statements about the overall strength of the evidence: “known to be toxic,” “probably toxic,” “possibly toxic,” “not classifiable,” or “probably not toxic.”
Grade the strength of the recommendations.

We were part of a team of scientists that developed the Navigation Guide method and applied steps 1–3 to the question “does developmental exposure to perfluorooctanoic acid (PFOA) affect fetal growth?” (Johnson et al. 2014; Koustas et al. 2014; Lam et al. 2014). Step 4 of the method, “grade the strength of the recommendations,” involves integrating the strength of the evidence on toxicity (from step 3) with information about exposure, the availability of less toxic alternatives, and patient values and preferences. This step was not addressed in the PFOA case study because of the limitations of our resources. Below we highlight the features of the method that are new to environmental health, features that differ from methods used in evidence-based medicine, a comparison of the results of the Navigation Guide method to previous reviews of PFOA exposure and toxicity, limitations of the Navigation Guide method, and future directions.

### Navigation Guide Features New to Environmental Health Reviews

To initiate the development of the Navigation Guide methodology, we convened a novel interdisciplinary team of 22 individuals from governmental and nongovernmental organizations and academia (Woodruff et al. 2011). Two members of this team, Daniel Fox (President Emeritus of the Milbank Memorial Fund) and Lisa Bero (currently Co-Chair of the Cochrane Collaboration) were world-renowned experts on systematic review methodologies used in the clinical sciences. Seven members were scientists or environmental health advocates from international, national, state, and local government agencies and a nongovernmental organization directly engaged in developing and/or employing strength-of-evidence conclusions in decision making on environmental chemicals: David Gee (European Environmental Agency), Vincent James Cogliano (IARC), Kathryn Guyton (U.S. EPA), Lauren Zeise (California Environmental Protection Agency), Julia Quint (California Department of Public Health, retired), Karen Pierce (San Francisco Department of Public Health), and Heather Sarantis (Commonweal). Eleven were health professionals with expertise in women’s, reproductive, pediatric, and/or environmental health: Jeanne Conry (American Congress of Obstetricians and Gynecologists District IX and Kaiser Permanente), Mark Miller (UCSF Pediatric Environmental Health Specialty Unit), Sarah Janssen (Natural Resources Defense Council), Beth Jordon and Rivka Gordon (Association of Reproductive Health Professionals), Sandy Worthington (Planned Parenthood Federation of America), Pablo Rodriguez (Brown Medical School and Women & Infants Hospital of Rhode Island), Michelle Ondeck and Judith Balk (University of Pittsburgh), Victoria Maizes (University of Arizona), and Ted Schettler (Science and Environmental Health Network). Finally, our own expertise has involved decades of work at the interface of environmental and occupational health and public policy. At the time of publication of the method, none of the collaborators reported a competing financial interest.

To conduct the first application of the Navigation Guide method, we assembled a team of nine scientists from academia and the U.S. EPA that encompassed the multidisciplinary expertise required to apply the methodology, including in environmental health sciences, epidemiology, toxicology, risk assessment, biostatistics, and the science of systematic reviews (Johnson et al. 2014; Koustas et al. 2014; Lam et al. 2014). One team member, Karen Robinson (Director of the Evidence Based Practice Center at Johns Hopkins University), was an expert on the identification, synthesis, and presentation of evidence for informing health care decisions and research; three team members, Patrice Sutton, Erica Koustas, and Paula Johnson had formal training in Cochrane and/or GRADE methodologies. None of the review team reported a competing financial interest.

The method developed and applied through these interdisciplinary teams builds on the best practices in research synthesis in evidence-based medicine and environmental health. Key points of departure of the Navigation Guide from current methods of expert-based narrative reviews in environmental health include the following.

**Figure 1:**
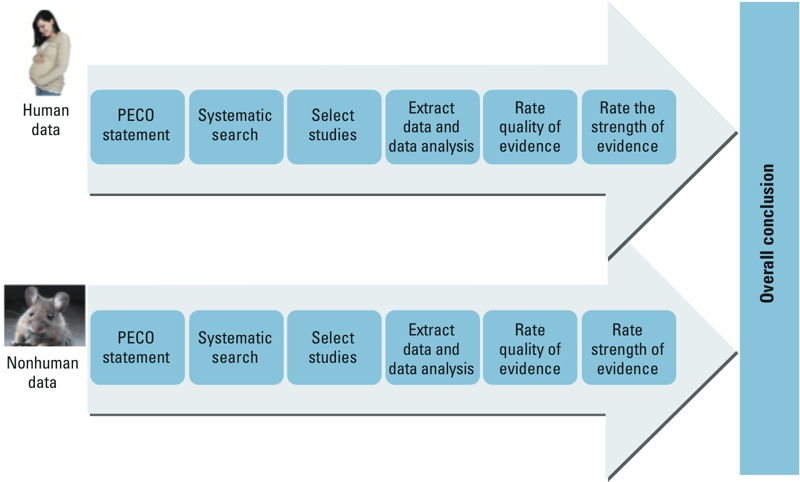
Steps in the Navigation Guide protocol. PECO, participants, exposure, comparator, and outcome(s).

1. *A protocol.* The application of the Navigation Guide is guided by a detailed protocol developed prior to undertaking the review (Figure 1). In contrast, expert-based narrative review methods do not provide a document that predefines a specific question to be answered and sets up the “rules” of the evaluation. A predefined protocol is a staple of systematic reviews in the clinical sciences because it reduces the impact of review authors’ biases, provides for transparency of methods and processes, reduces the potential for duplication, and allows for peer review of the planned methods (Higgins and Green 2011). Notably, the protocol also provides a transparent forum to incorporate the expertise of nonscientists, including health-impacted populations and their advocates, in framing a meaningful study question. The protocol is developed around a “PECO” statement [participants, exposure, comparator, and outcome(s)], which provides the framework from which studies are identified and selected for inclusion. The PECO statement is similar to recommendations by the National Academy of Sciences for improving the design of risk assessment through planning, scoping, and problem formulation to better meet the needs of decision makers (NRC 2009).

2. *Standardized and transparent documentation including expert judgment.* Systematic reviews are not “automated” or “computerized” or otherwise conducted without applying judgment (Guyatt et al. 2011). The fundamental shift from existing methods of expert review in environmental health science is that each step of the Navigation Guide is conducted in a thorough, consistent, and transparent manner, and all information, including judgments, is documented and displayed in the same way. In short, the rationale for a decision is traceable, reproducible, and comprehensible.

3. *Assessment of “risk of bias.”* The assessment of “risk of bias,” defined as characteristics of a study that can introduce systematic errors in the magnitude or direction of the results (Higgins and Green 2011), is a new concept in environmental health. Systematic review methodologies distinguish between study-quality criteria that can introduce a systematic error in the magnitude or direction of the result (i.e., risk of bias or “internal validity”) from other methodological quality or reporting elements, which are related to important standards by which a study is conducted (e.g., adherence to human subjects and animal welfare requirements) or reported (e.g., complete information provided), but that do not systematically influence study outcomes. A study conducted to the highest methodological standards can still have important risk of bias that will affect the magnitude or direction of a study outcome.

Risk of bias domains have been well developed and empirically shown to influence study outcomes in experimental human studies (Higgins et al. 2011; Roseman et al. 2011). However, risk of bias domains that are equally agreed upon for human observational studies are lacking. In the PFOA case study, we based our risk of bias domains for observational human studies on the domains used by the Cochrane Collaboration and the Agency for Healthcare Research and Quality (Higgins and Green 2011; Viswanathan et al. 2012), including recruitment strategy, blinding, confounding, incomplete outcome data, selective reporting, and exposure assessment.

Domains for risk of bias for animal studies are also under development. Although 30 instruments have been identified in the environmental health literature for evaluating the quality of animal studies, they are mostly composed of domains related to reporting requirements, such as compliance with regulatory requirements, description of the statistical model, and test animal details; importantly, they do not include all the risk of bias domains in use in human experimental studies (Krauth et al. 2013).

To develop risk of bias domains for applying the Navigation Guide to animal studies, we adapted the risk of bias domains used in human experimental studies that have an empirical basis, including *a*) sequence generation, *b*) allocation concealment, *c*) blinding, *d*) incomplete outcome data, and *e*) selective reporting [see Figure 1 in Johnson et al. (2014) and Figure 1 in Koustas et al. (2014)]. According to GRADE, these five criteria address nearly all issues that bear on the quality of human experimental evidence (Balshem et al. 2011). Further, these elements have been shown in the preclinical animal literature to influence study outcomes (Vesterinen et al. 2010). Our rationale was that risk of bias in a nonhuman experiment is comparable to risk of bias in human and preclinical animal experiments.

Further, in both human and animal studies, we included a “conflict of interest” risk of bias domain. This domain has been proposed—but not yet adopted—by Cochrane and GRADE as an important risk of bias (Bero 2013). This is based on empirical data from studies of the health effects of tobacco (Barnes and Bero 1997, 1998), the safety and efficacy of pharmaceuticals (Bero et al. 2007; Lexchin et al. 2003; Lundh et al. 2012), and medical procedures (Popelut et al. 2010; Shah et al. 2005), which have all shown that, on average, source of funding influences study outcome.

The assessment of risk of bias in the PFOA case study revealed worrisome truths about the conduct and reporting of experimental animal studies in environmental health. In particular, we found that included toxicological studies uniformly did not apply methodological approaches that are empirically recognized as minimizing bias in human experimental study outcomes. In particular, none of the studies reported how or if they used adequate allocation concealment, regardless of whether the studies were conducted through Good Laboratory Practices (GLP), by industry groups, or by independent research laboratories. Suboptimal experimental animal study design and reporting is prevalent in the preclinical literature, and introduces bias into study findings (Bebarta et al. 2003; Landis et al. 2012; Macleod et al. 2004; McPartland et al. 2007; van der Worp and Macleod 2011; van der Worp et al. 2007; Vesterinen et al. 2011). For example, studies by the Collaborative Approach to Meta-Analysis and Review of Data from Experimental Studies (CAMARADES) collaboration have shown that studies using randomization and allocation concealment reported less improvement in heart response measures in animal models of focal ischemia treated with the pharmaceutical NXY059 (Macleod et al. 2008) and less improvement in neurobehavioral scores in animal models of intracerebral hemorrhage (Frantzias et al. 2011) than other studies.

In our outreach efforts related to the Navigation Guide, we found that environmental health researchers in many and varied settings reported that methodological approaches to reduce bias in toxicological studies were not widely recognized or were not customary practices.

A second challenge to conducting risk of bias assessments and quantitative analyses in the PFOA case study was that the necessary data were not all reported in the published studies. Our efforts to contact study authors to get the needed data were moderately successful [i.e., 18 of 28 (64%) authors that were contacted responded] and were critical to our ability to conduct the review. We anticipate that contacting study authors will be a necessary step for those conducting systematic reviews until such time that steps are undertaken—by journals, funding agencies, and through study registries—to standardize optimal reporting. Our findings underscore the urgency of calls for improved access to the data needed to conduct scientifically robust reviews of environmental health science (Goldman and Silbergeld 2013) and the importance to environmental health of nascent efforts in the preclinical arena to develop improved experimental animal study design and reporting (Landis et al. 2012; van der Worp and Macleod 2011; Vesterinen et al. 2011).

4. *Comprehensive and efficient search strategy.* The outcome of the Navigation Guide search method demonstrated the potential for systematic reviews to be more comprehensive than traditional reviews. We evaluated four more human studies than did an expert panel appointed to review the health effects of PFOA (C8 Science Panel 2011). The search strategy used to gather data for the C8 panel was not published. However, because these four papers did not present data that proved to be essential to the conclusions of the review (i.e., the data included were from small studies that did not weight heavily in the meta-analysis), they could have been identified by the C8 Panel’s search but excluded from their reference list. Our comprehensive search strategy captured studies that measured PFOA exposure and fetal growth parameters but did not necessarily draw associations between the two. The four additional studies included in our review did not have birth weight or other fetal growth measures as the primary outcome or main topic of the paper (Fromme et al. 2010; Kim S et al. 2011; Kim SK et al. 2011; Wang et al. 2011). However, because our search identified these studies, we included them, contacted the study authors, and obtained additional relevant data to support our review from authors of two of these studies (Fromme et al. 2010; Kim S et al. 2011) and were referred by one author (Wang et al. 2011) to an article under peer review at the time on the same cohort with more relevant data (Chen et al. 2012). We also identified 10 more nonhuman studies than were included in our own earlier nonsystematic literature review. Our adoption of a search filter for animal studies in use in the preclinical literature (Hooijmans et al. 2010) greatly expedited the development of a search for relevant animal studies.

We found that casting a wide net for relevant studies was feasible because of the development of a PECO statement from which we developed very explicit criteria used to efficiently screen titles and abstracts and because of the use of a software program that expedited the screening process. For this case study, our search strategy identified slightly more than 2,000 nonhuman and 3,000 human potentially relevant studies. For the human data, it took 1 person-day to screen titles and abstracts (resulting in 248 articles eligible for full text review) and 1 week to do a full text review, which identified 18 relevant studies for evaluation. The time for the evaluation of the nonhuman data was similar.

Further, by applying a method that seeks to extract the exact same information, laid out in the same transparent way, our ability to interpret and understand the results was straightforward. As the application of systematic reviews expands, we anticipate greater efficiencies will be gained, for example, through the development of improved search filters and screening and management systems.

5. *Separation of the science from values and preferences.* The PFOA case study demonstrated steps 1–3 of the Navigation Guide methodology, the result of which was a concise statement regarding PFOA’s toxicity. However, toxicity is just one aspect of a risk management decision in environmental health. In step 4 of the Navigation Guide, which is modeled after GRADE’s methods for rating treatment recommendations (Guyatt et al. 2008a), other important factors are brought to bear on recommendations for prevention, including values and preferences, extent of exposures, the availability of safer alternatives, and costs and benefits. Thus, the Navigation Guide transparently and explicitly delineates the science from other key considerations. Although we did not have the resources to operationalize step 4 in the PFOA case study, we hope to do so in future case studies.

### Navigation Guide Features Different from Evidence-Based Medicine

Because of differences between environmental and clinical health sciences related to the evidence base and decision context, systematic review methodologies used in the clinical sciences were not seamlessly applicable to environmental exposures (Woodruff et al. 2011). Two key points of departure of the Navigation Guide methodology from evidence-based medicine are as follows.

1. *The body of human observational studies is assigned a “moderate” quality rating.* The Navigation Guide assigns *a priori* a “moderate” quality rating to the body of human observational evidence. This initial quality rating of “moderate” is independent of the specifics of the studies in the assessment. The actual quality of the body of human observational studies is then accounted for through upgrading or downgrading the “moderate” rating based on *a priori* criteria. In contrast, systematic reviews in the clinical sciences, which proceed from the availability of human experimental evidence, assign an *a priori* rating to the body of human observational studies of “low” quality. In particular, Cochrane and GRADE have been developed primarily based on evaluation of randomized controlled clinical trials (RCTs), and in this context, relative to RCTs, GRADE considers human observational studies to be “low”-quality evidence (Balshem et al. 2011).

Our rationale to assign the body of human observational studies a rating of “moderate” and not “low” quality was based on the absolute and relative merit of human observational data in evidence-based decision making in environmental and clinical health sciences. Overall, human observational studies are recognized as being a reliable source of evidence in the clinical sciences because not all health care decisions are, or can be, based on RCTs. The contribution of observational studies to certain health care decisions is underscored by the conclusion of a 2008 Institute of Medicine (IOM) panel, which found observational studies to be the preferred method for evaluating the causes of disease, which would include the contribution of environmental agents. The IOM panel noted that observational and experimental studies each can provide valid and reliable evidence, with their relative value dependent on the clinical question (IOM 2008). In this context, the IOM report (IOM 2008) stated that

Observational studies are generally the most appropriate for answering questions related to prognosis, diagnostic accuracy, incidence, prevalence, and etiology.

Moreover, recognition of the absolute value of human observational data to evidence-based clinical decision making is increasing. There are several reasons for this. For example, the speed and complexity with which new medical interventions and scientific knowledge are being created make it unlikely that the evidence base required for treatment and cost-effective health care delivery across subpopulations can be built using only RCTs (Peterson 2008). It is also expected that electronic medical records will revolutionize medical research by facilitating comprehensive, longitudinal observational data in an instant (Halvorson 2008). Finally, ethical considerations virtually preclude experimental human data from the environmental health evidence stream. Therefore, relative to the evidence available for decision making in environmental health, human observational studies are the “gold standard” of the evidence base.

2. *Diverse evidence streams are combined. In vitro*, *in vivo, in silico*, and human observational studies all inform decision making on environmental chemical exposures. However, there is currently no agreed-upon standard method in clinical medicine for evaluating evidence simultaneously across disparate evidence streams. We therefore adapted a mixture of IARC’s method for integrating human and nonhuman evidence (IARC 2006) linked to strength of evidence descriptions in use by the U.S. EPA (1991, 1996). Although this transparently produced a clear, concise, and recognizable bottom line (i.e., “known to be toxic,” “probably toxic,” “possibly toxic,” “not classifiable,” or “probably not toxic”), further development of precise criteria, definitions, and nomenclature for strength of evidence that meets the needs of a wide range of decision makers will be an important undertaking as uptake of methodology moves forward.

### Comparison of the Navigation Guide Method to Previous Reviews of PFOA and Fetal Growth

The authors of the review conducted with the Navigation Guide methodology concluded that “developmental exposure to PFOA adversely affects human health based on sufficient evidence of decreased fetal growth in both human and nonhuman mammalian species” (Lam et al. 2014). To compare these results to previous reviews, we searched PubMed (http://www.ncbi.nlm.nih.gov/pubmed) without date or language restrictions for reviews of “PFOA” or “pefluorooctanoic acid.” Of the 48 papers identified, 12 included discussions of reproductive or developmental health. Two additional reviews (Butenhoff et al. 2006; Stahl et al. 2011) were identified at the time we were embarking on this project, and we also included those publications. Of 14 reviews, all but 1 (Stahl et al. 2011), which was not indexed in PubMed, were also identified by our search strategy for the PFOA case study (Johnson et al. 2014).

Table 1 compares the 14 reviews of PFOA exposure and toxicity identified by our search to seven key features of systematic and transparent review methods, that is, Cochrane and GRADE. All 14 reviews were conducted using nonsystematic, expert-based narrative methods. Of the 14 reviews, 13 defined a study question, 9 included a summary of findings table, 3 specified criteria for included studies, 2 included limited information about their search strategy, 2 conducted data analysis, and 1 assessed the quality of individual studies. None of the 14 reviews systematically or transparently assessed risk of bias for individual studies, and none integrated human and nonhuman evidence to produce an overall summary of the strength of the evidence (Butenhoff et al. 2004; DeWitt et al. 2009; Hekster et al. 2003; Jensen and Leffers 2008; Kennedy et al. 2004; Kudo and Kawashima 2003; Lau et al. 2004, 2007; Lindstrom et al. 2011; Olsen et al. 2009; Post et al. 2012; Stahl et al. 2011; Steenland et al. 2010; White et al. 2011). These 14 reviews either produced vague or indeterminate answers to the question of PFOA’s toxicity, or presented a clear answer (i.e., “PFOA is a known developmental toxicant”) (White et al. 2011) without specifying the search methods, study inclusion criteria, or statistical methods that produced the answer. Our comparison of the methods and results of these narrative reviews to the Navigation Guide method demonstrated that the application of the Navigation Guide provided more transparency about the steps taken in the review and a consistent path to a clear answer compared with the methods of expert-based narrative review that are currently employed in environmental health. Our results demonstrated that improved methods of research synthesis under development at the National Toxicology Program (Birnbaum et al. 2013; Rooney et al. 2014) and under consideration by the U.S. EPA (NRC 2011, 2014a, 2014b; U.S. EPA 2013a) are fully achievable.

**Table 1:** Comparison of the PFOA reviews’ methods according to key features of Cochrane and GRADE systematic and transparent review methods.

Reference	Specify study question	Specify inclusion/exclusion criteria	Conduct reproducible search	Assess “risk of bias”	Data analysis and/or meta-analyses	Summary of findings table	Assess quality and strength of body of evidence
Navigation Guide PFOA case study 2014^*a*^	Yes	Yes	Yes	Yes	Yes	Yes	Yes
Post et al. 2012	Yes	No	No	No	Some data analysis (BMD, BMDL)	Yes	No
Lindstrom et al. 2011	Yes	No	No	No	No	No	No
Stahl et al. 2011	Yes	No	No	No	No	Yes	No
White et al. 2011	Yes	No	No	No	No	Yes	No
Steenland et al. 2010	Yes	No	No	No	No	Yes	No
DeWitt et al. 2009	Yes	No	No	No	No	No	No
Olsen et al. 2009	Yes	Inclusion criteria	No	No	No	Yes	Assess methodological weaknesses of included studies
Jensen and Leffers 2008	No	No	No	No	No	No	No
Lau et al. 2007	Yes	No	No	No	No	Yes	No
Butenhoff et al. 2004	Yes	Yes	No	No	Some data analysis (MOE, LBMIC_10_)	Yes	No
Kennedy et al. 2004	Yes	No	No	No	No	Yes	No
Lau et al. 2004	Yes	No	Limited discussion of literature search	No	No	No	No
Hekster et al. 2003	Yes	Some inclusion criteria described in cited report by same authors	Limited discussion of literature search	No	No	Yes	No
Kudo and Kawashima 2003	Yes	No	No	No	No	No	No
Abbreviations: BMD, benchmark dose; BMDL, BMD lower confidence limit; LBMIC_10_, lower 95% confidence limit of a modeled 10% response; MOE, margin of exposure. ^***a***^Data presented by Johnson et al. (2014), Koustas et al. (2014), and Lam et al. (2014).							

### Limitations

A limitation of the Navigation Guide systematic review method is that although its overall architecture is based on empirically proven and/or time-tested methods (i.e., methods in use by Cochrane, GRADE, IARC, and the U.S. EPA), novel aspects of the method need further development and validation, including *a*) rating the quality and strength of nonmammalian animal and *in vitro* and *in silico* evidence streams; *b*) reaching a consensus on risk of bias domains for human observational studies and nonhuman studies; *c*) developing well-defined, measurable evidentiary bars for the factors used to downgrade the quality of environmental health evidence (i.e., indirectness, inconsistency, imprecision, and publication bias) and for upgrading human evidence (i.e., dose response, large magnitude of effect, and confounding minimizes effect); and *e*) exploring whether it makes a difference to the final quality rating if we assign the entire body of human observational studies a “moderate” rating and then downgrade for lesser quality study designs, or, as proposed in the NTP’s framework, we assign different types of human observational studies different ratings from the start (i.e., cross-sectional studies, case–control studies, and case series or reports are rated as “low” quality, and cohort and nested case–control studies are rated as “moderate” quality) (Rooney et al. 2014). Improved statistical tools for data analysis and integration will also advance the application of systematic review methods in environmental health. Whether the use of our nomenclature for the final strength of evidence ratings (i.e., “known to be toxic,” “possibly toxic,” and so on) will be useful to decision makers is also untested, and consensus methods for classifying strength of evidence for noncancer health outcomes is a critical research and policy need (Gee 2008).

In addition, the application of the Navigation Guide method—just like any expert-based narrative review—can be poorly executed. For example, a systematic review can be conducted that does not specify a study question relevant to decision making, or an incomplete search strategy can fail to uncover information pertinent to the review. However, a poorly performed systematic review is more readily detected because the methods are transparently displayed.

The capacity for improved methods of research synthesis in environmental health to spur timely health protective decision making is also limited by the shortcomings of the available evidence stream that is produced by current systems of generating scientific knowledge. One key example is the need for an unconflicted underlying evidence stream. As the Deputy Editor (West) of *JAMA* observed in 2010, “the biggest threat to [scientific] integrity [is] financial conflicts of interest” (Rennie 2010). Moreover, risk of bias assessments leave unaddressed the inherent biases in environmental health science methodologies that generate false negatives and rely on strength of evidence criteria that are unequal to the task of addressing complex and multicausal disease etiologies (Gee 2008). Finally, there are many other formidable nonscientific, social, and political barriers to prevention-oriented action (European Environment Agency 2013; Michaels 2008).

### Future Directions

Shortening the time between scientific discovery and the prevention of exposures to toxic environmental chemicals is inextricably linked to the success of private and public sector efforts to advance safer and sustainable alternatives to toxic chemicals. The assessment of toxicity is an essential underpinning of such efforts (Edwards 2009; Malloy et al. 2013; Matus et al. 2012; Park et al. 2014; U.S. EPA 2012). As such, the Navigation Guide methodology has broad applicability to support efforts by businesses, governments, and consumers to compare and choose among various chemicals using a standardized and rigorous method.

As in the clinical application of systematic reviews, development of systematic and transparent methods of research synthesis in environmental health will be an ongoing process. Some immediate methodological needs relate to how to routinely integrate critical concepts into the interpretation of data, including low-dose effects, concordance in response across species, and human variability (including age and comorbidities). These issues were considered in the PECO question and statistical analyses of the PFOA case study, but a more thorough and overarching framework for how to integrate these concepts in systematic reviews is still needed. For example, failure to use animals with clinically relevant comorbidities, such as hypertension in stroke models, has been shown to bias the assessment of drug efficacy (Macleod et al. 2008; Sena et al. 2010), and we would expect that including animals with chronic conditions may affect findings for environmental chemicals. Robust methods to assess publication bias in environmental health science are also a need, because researchers can have financial and or other conflicts that can promote bias in opposite directions.

Uptake of methods of systematic and transparent review represents a new way of doing business in environmental health sciences. A realistic starting place is to recognize the potential for many or all of the challenges related to using systematic reviews in clinical medicine (i.e., perceived threats to physician autonomy, patient choice, and so on) to become our challenges. We will need to overcome a lack of knowledge of environmental health science and research synthesis methods by every key target audience. The application of systematic reviews in environmental health is inherently an interdisciplinary “team science” undertaking, and success will require formalizing the necessary expertise and assembling and training review teams in these new methods and relevant communication skills.

## Conclusion

Systematic and transparent methods of research synthesis are empirically based and can serve as a roadmap to more efficient and transparent decision making using the available data. The use of systematic review methods allows decision makers to act on any quality of evidence and in any direction. Moreover, the use of systematic reviews can prevent wasteful expenditures on studies that are duplicative or otherwise unnecessary for decision making (Chalmers and Glasziou 2009).

In his 1965 address to the Royal Society of Medicine, Sir Austin Bradford Hill, the statistician who pioneered the RCT, admonished his audience that while science is always incomplete and subject to change,

[it] does not confer upon us a freedom to ignore the knowledge we already have, or to postpone the action that it appears to demand at a given time. (Hill 1965)

Hill (1965) emphasized that “strong evidence” does not imply “crossing every ‘t’, and swords with every critic, before we act.” He proposed differential standards of evidence for different actions, a recommendation echoed by the National Academy of Sciences a half-century later in *Science and Decisions: Advancing Risk Assessment* (NRC 2009).

Because systematic review methods transparently distinguish between science, values, and preferences, they can help sharpen the terms of debates regarding whether we strive for more precision or more decisions about the meaning of the science to health.

This first case study of the Navigation Guide methodology demonstrated the successful application of a systematic and rigorous method for research synthesis designed to optimize transparency and reduce bias in the evaluation of environmental health information. Government agencies can use the Navigation Guide methodology to craft evidence-based statements regarding the relationship between an environmental exposure and health (steps 1–3). Government agencies called on to make risk management decisions can also apply step 4 of the Navigation Guide to grade the strength of recommendations for prevention. Professional societies, health care organizations, and other potential guideline developers working with toxicologists can use the Navigation Guide to craft consistent and timely recommendations to improve patient, and ultimately population, health outcomes (steps 1–4). The institutionalization of robust methods of systematic and transparent review would provide a concrete mechanism for linking science to timely action to prevent harm. Although simple in concept, Navigation Guide methodology will require sustained visionary leadership harnessed to substantive investment as well as the intellectual curiosity and commitment of environmental and clinical health scientists and advocates.
